# Mobile Fotonovelas Within a Text Message Outreach: An Innovative Tool to Build Health Literacy and Influence Behaviors in Response to the COVID-19 Pandemic

**DOI:** 10.2196/19529

**Published:** 2020-08-10

**Authors:** Rena Brar Prayaga, Ram S Prayaga

**Affiliations:** 1 mPulse Mobile, Inc Encino, CA United States

**Keywords:** text messaging, SMS, mobile fotonovelas, COVID-19, social isolation, social support, health behaviors, health literacy, health plans

## Abstract

With all 50 US states reporting cases of coronavirus disease (COVID-19), people around the country are adapting and stepping up to the challenges of the pandemic; however, they are also frightened, anxious, and confused about what they can do to avoid exposure to the disease. Usual habits have been interrupted as a result of the crisis, and consumers are open to suggestions and strategies to help them change long-standing attitudes and behaviors. In response, a novel and innovative mobile communication capability was developed to present health messages in English and Spanish with links to fotonovelas (visual stories) that are accessible, easy to understand across literacy levels, and compelling to a diverse audience. While SMS text message outreach has been used to build health literacy and provide social support, few studies have explored the benefits of SMS text messaging combined with visual stories to influence health behaviors and build knowledge and self-efficacy. In particular, this approach can be used to provide vital information, resources, empathy, and support to the most vulnerable populations. This also allows providers and health plans to quickly reach out to their patients and members without any additional resource demands at a time when the health care system is severely overburdened.

## Background

Coronavirus disease (COVID-19) has profoundly changed our experience of everyday life and our interpersonal connections. Both the US Centers for Disease Control and Prevention (CDC) and World Health Organization have recommended precautions to prevent and slow transmission of the disease; these include washing hands frequently with soap and water for 20 seconds, covering coughs and sneezes, and avoiding handshakes [1,2]. At the same time, social distancing and stay-at-home orders have required people to stay home and leave only for essential activities; this can lead to increasing social isolation and loneliness, especially for the 35.7 million Americans (28% of households) who live alone [3].

## Health Literacy and Fotonovelas

Health literacy is the degree to which individuals can obtain, process, and understand basic health information and the services needed to make appropriate health decisions [4]. Approximately 80 million Americans have low health literacy; this has been associated with disparities in health care access, lower use of health care services, and poorer health outcomes [5]. Limited or insufficient health literacy has also been associated with lower adoption of protective behaviors, such as vaccinations, hand hygiene, and other self-care measures [6]. The urgent need to avoid exposure to COVID-19 would require a change in habits and behaviors, such as washing hands more frequently and maintaining a safe physical distance from others outside the home. It has been suggested that SMS text message campaigns for public health are the “most effective medium for mass dissemination due to their reach, immediacy, opportunity for data collection and personalization, ability to tailor and adapt information, and opportunity to link to other sources [7].” Our goal was not only to use SMS text messages as a rapid deployment tool but also to build health literacy by including a visual story within the text messages that shows people modeling healthy behaviors to protect against the virus. While several innovative technologies have been developed to improve health literacy, a review of the literature revealed a lack of visual tools or mobile fotonovelas within text messages as a strategy to build health literacy and influence health behaviors. 

There is substantial evidence that text messaging can be used for health outreach and to influence health behaviors [8]. At the same time, fotonovelas, a traditional print medium originally developed for Latin American audiences, have been used with great success with participants from other (eg, Dutch) cultures, particularly among low-literacy or underserved populations [9-11]. Similar to a comic book in format, fotonovelas typically tell a story using a dramatic or soap opera–style plot with illustrations or photographs and dialogue bubbles to capture the user's attention and share an important lesson. They have been used to help patients understand the value of preventive care and screenings and to improve self-management of chronic conditions. They are also more likely to be passed on to family or friends through social networks and can increase their reach beyond the targeted individual.

In this case, we wanted to adapt the traditional print format by creating a series of mobile (digital) fotonovelas or illustrated comic strips that could be used to improve health literacy around COVID-19, to fill knowledge gaps around perceived severity and susceptibility, and to help health plan members build new healthy habits to avoid exposure to the virus. We started with two mobile fotonovelas (in both English and Spanish) to (1) build health literacy and awareness about simple changes in daily habits that could make a great difference in keeping safe, and (2) provide support to address the challenges of staying at home and social isolation as communities attempt to lower the risk of infection and “flatten the curve.” These fotonovelas were delivered to health plan members and patients as a link within SMS text messages. SMS is an obvious and well-accepted channel for rapid deployment that has a high rate of adoption; it seemed particularly appropriate given the urgent need to communicate health risks about the virus. The text messages with links to fotonovelas were designed to be educational, with lighthearted content, and to reduce cultural and linguistic barriers. The readability of all content was at or below a sixth grade reading level.

## Fotonovela 1: Promoting Healthy Behaviors to Prevent the Spread of COVID-19

**Figure 1:**
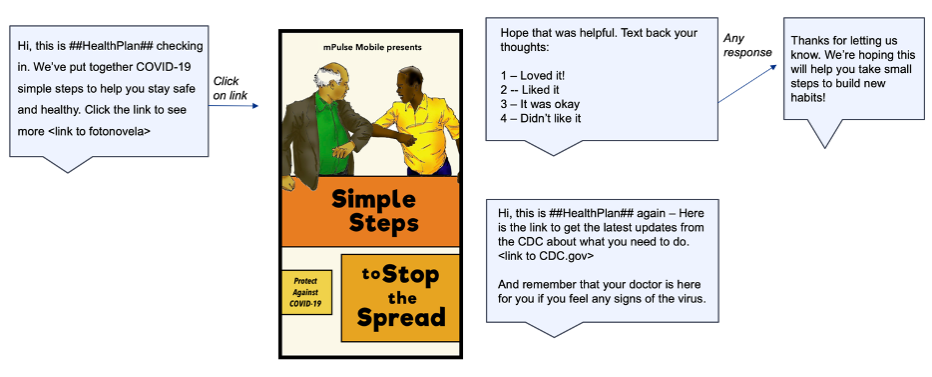
Text workflow introducing Fotonovela 1.

**Figure 2:**
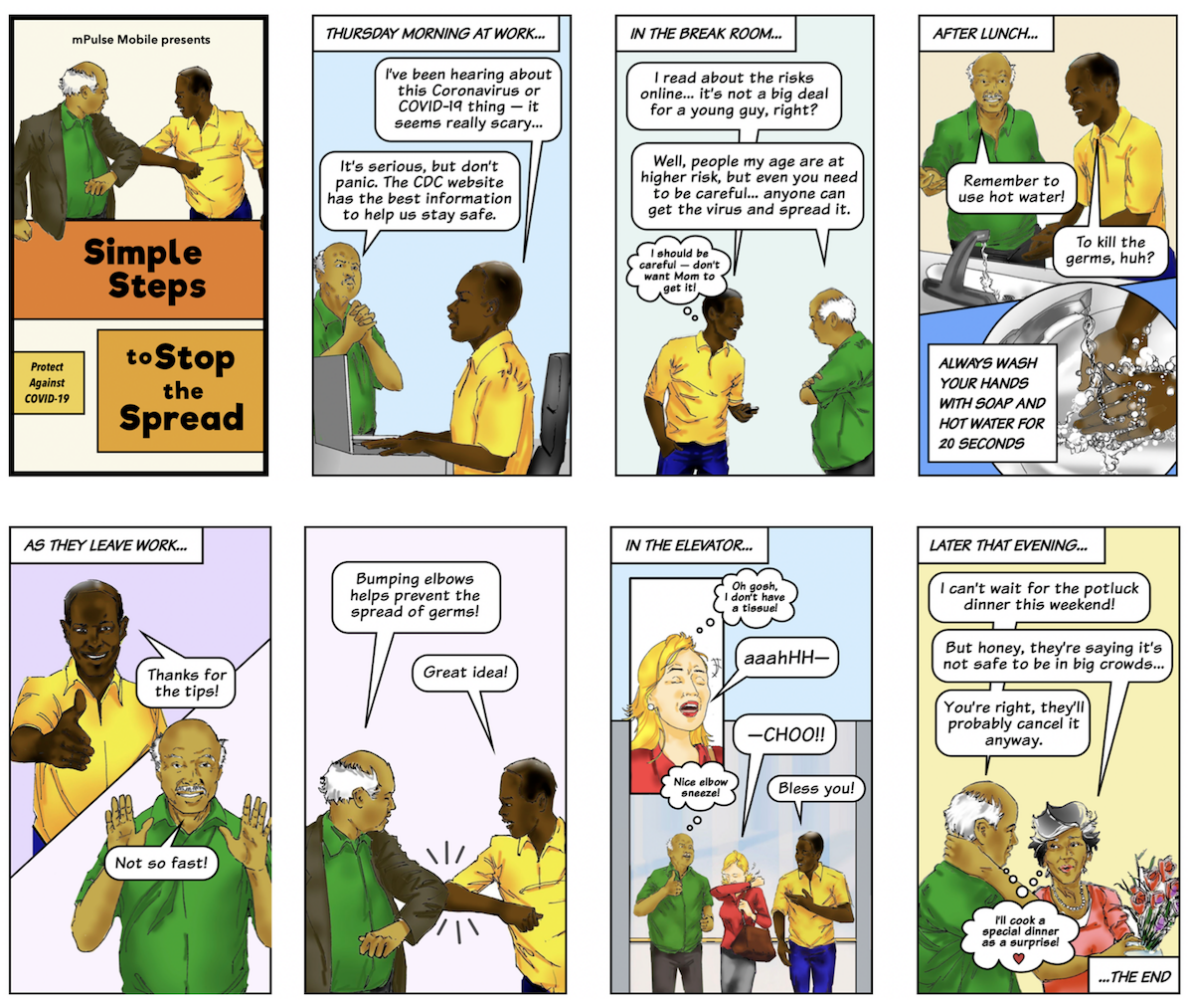
Images from Fotonovela 1: Simple Steps to Stop the Spread.

The messages in Fotonovela 1 were created as an early response to the COVID-19 threat and were informed in part by the Health Belief Model (HBM) [12-14], which was originally developed to study patient screening behavior for tuberculosis and is commonly used to drive behaviors relating to health promotion and disease prevention. In this case, recipients received an SMS text message telling them that their health plan had put together “COVID-19 simple steps” to help them stay safe and healthy, and they were asked to click on a link to see more. Upon clicking, they viewed the mobile fotonovela, which consisted of 8 frames in story format showing different settings and how to stay safe around people in those settings. The various scenes in the story discussed high-risk individuals, washing hands, elbow bumps, elbow sneezes, using only trusted information sources on the internet, and avoiding crowds (see Figures 1 and 2).

We were addressing the various determinants of behavior or constructs that are outlined in the HBM and that are likely to influence preventive behaviors: perceived severity (Scene 1: how bad is the virus), perceived susceptibility (Scene 2: does it really apply to me and how do my actions impact others I care about), perceived benefits (Scene 3: washing hands will help keep me safe), perceived barriers to action (Scenes 4 and 5: but I have to change my behavior and stop shaking hands, be more careful about sneezing, and engage in social distancing), and exposure to factors that prompt action (Scene 6: influencing each other to avoid crowds and stay home). The goal of this type of mobile fotonovela is to build self-efficacy (the efficacy to influence events in one’s life) [15] so that recipients feel empowered to pursue strategies and develop new habits that are likely to be successful in addressing the perceived threats or challenges posed by COVID-19. We also relied on social cognitive theory [16], which posits that understanding health risks and benefits will influence changes in health habits and behaviors if an individual believes that these new health behaviors can positively impact their health. As part of this process, there is an evaluation of possible benefits and losses and social approval or disapproval associated with new behaviors. By using a story format, we were able to (indirectly) share important messages, build empathy and understanding, and avoid making the recipient feel defensive or detached. The focus on simple steps made the changes feel easier, and these small successes could build self-efficacy and confidence over time.

## Fotonovela 2: Emphasizing the Importance of Staying Home to Save Lives

As the situation evolved, it became clear that we would need to provide support for extended periods of physical distancing (also called “social distancing”) to slow the spread of COVID-19. An additional challenge was posed by social isolation as a result of restricted movement outside the home due to self-imposed or mandatory precautionary measures. There is much evidence that social isolation (or a lack of social interaction) can have adverse health effects and even reduce life expectancy when it is experienced as loneliness. Social isolation increases the risks of cardiovascular disease and cognitive decline, and it even weakens the immune system. A recent study suggested that the health effects of social isolation are as damaging as smoking 15 cigarettes a day, and this effect is particularly noticeable among older people [17]. Social isolation in the era of COVID-19 adds an additional level of uncertainty, anxiety, and fear that can quickly influence quality of life and well-being and start to impact mental and physical health. In response, we created Fotonovela 2, which focuses on staying home whenever possible, maintaining healthy physical separation from others outside the home, developing strategies to remain balanced and positive despite limited social contact, and finding ways to remain emotionally connected with friends and family using technology. The messages in Fotonovela 2 address the effects of limited social contact over several weeks and the impact of these restrictions on personal well-being and perceived loneliness.

There is strong evidence from studies of psychology and behavioral economics [18] that people tend to engage in cognitive biases as part of the decision-making process, in which they assess the probability of uncertain events using heuristic principles that may contain severe errors. One such cognitive bias or illogical heuristic is *optimism bias* [19], where people underestimate risks when considering potential harm in the form of disease or catastrophe and also (erroneously) tend to believe that others are more likely to be impacted than themselves. This is coupled with *present bias* [20], which tends to overvalue immediate rewards in the present (seeing friends at a get-together in two days) at the expense of long-term benefits (putting off the get-together so that everyone reduces their likelihood of contracting COVID-19). We believe that the prolonged nature and uncertain term of the stay-at-home requirements will only exacerbate these biases. To address both present bias and optimism bias, the consequences of not engaging in physical distancing are made particularly salient through the “Stay home, save lives” caption, which mirrors the messaging being promoted by the CDC.

**Figure 3:**
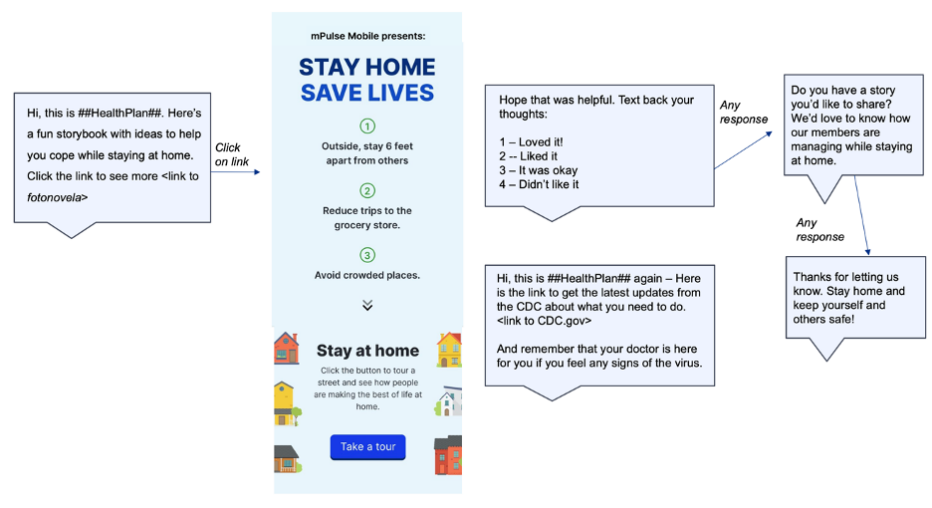
Text workflow introducing Fotonovela 2.

**Figure 4:**
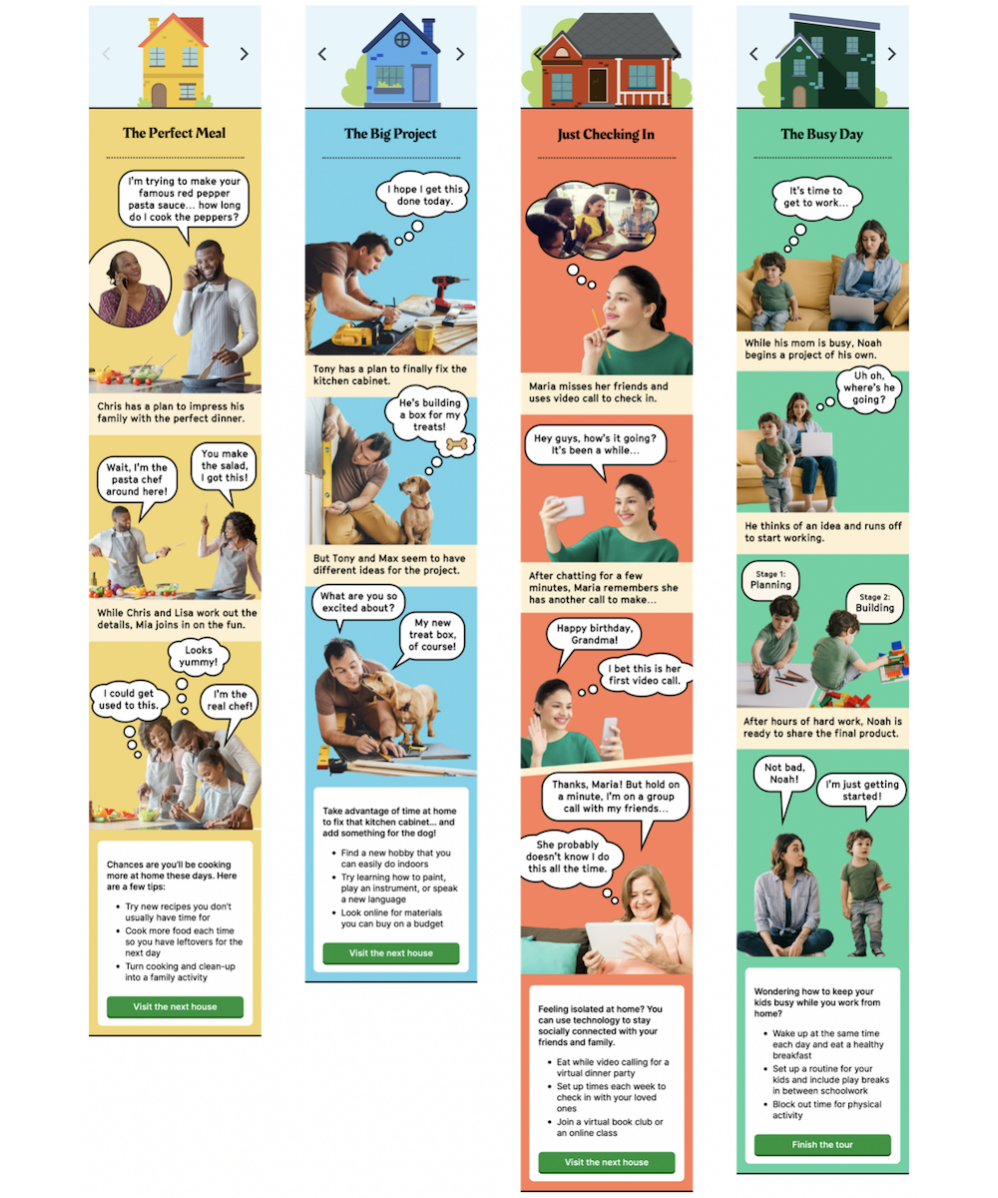
Images from Fotonovela 2: Stay Home, Save Lives.

Fotonovela 2 (see Figures 3 and 4) shows us four houses in a neighborhood and introduces us to the people in these homes. As the user scrolls down, a story unfolds behind closed doors and shares how people in the neighborhood are staying at home, coping with change, and finding ways to stay emotionally connected and positive. At the end of each story, there are specific suggestions to encourage users to explore new ways to stay actively engaged and develop a sense of routine even while being confined within their homes. By infusing the scenes with everyday examples of people making changes, expressing empathy, and staying positive, a secondary goal is established to build resilience and hope at a time of deep uncertainty. The primary goal is to overcome these biases, tap into prospect theory and loss aversion [21], and update risk assessment calculations so that individual decision-making and behavior more closely follow normative guidelines.

For Fotonovela 2, we used photographs instead of illustrations, presenting images of real people against simple backgrounds with thought and speech bubbles. This visual presentation is more similar to that of traditional printed (or paper-based) fotonovelas and presented the stories within each vignette with elements of humor and lightheartedness (a dog that speaks or a toddler who has a design plan). The expectation is that the audience can relate to the situation presented in the first frame (the problem) but also can gain from the strategy offered in the subsequent frames (the solution). While each vignette ends with core points of the solution and related action suggestions, we rely on the visual story to achieve the communication objectives: to convey a sense of togetherness, address doubts and apprehensions, and provide concrete and positive ways to cope and build a sense of agency.

In addition to the fotonovelas, we created a series of check-ins via SMS text messages with advice, suggestions, and support on a range of topics, including cooking ideas, exercising at home, importance of a daily routine, following a regular sleep schedule, suggestions to manage stress and increase mindfulness, and avoiding monitoring news reports throughout the day. The messages included links to helpful resources and were crafted to provide a lighter and more positive tone to help people reshape or reframe their day-to-day routines, even as they felt more cut off from social interactions and experienced high levels of uncertainty about the future.

While Fotonovela 2 and the related text messages do not explicitly discuss COVID-19, focusing instead on supportive and empathetic content, we built an extensive natural language understanding (NLU) system that can recognize and address member questions and concerns relating to COVID-19. For example, if a member asked “How do I know if I have coronavirus?”, the NLU system would understand the question as “symptom-related” and automatically respond with a message pointing the member to the appropriate authoritative sources (eg, the CDC website, health plan website, or state website). This feedback system also allows us to add new content based on topics that were not included in the original program.

## Early Feedback and Future Considerations

The mPulse Mobile platform delivers text messages to patients and members on behalf of health care companies. The platform consists of several components that collectively enable companies to interactively engage with their end users about appointments, refills, gaps in care, or other health-related topics [22]. This was our first use of a mobile fotonovela to share important health information to address health beliefs, build self-efficacy, and influence health behaviors. The characters in the visuals are culturally diverse, vary in age and gender, and communicate in English or Spanish. We were excited to find that this approach was effective in reaching over 100,000 health plan members across the age spectrum (as old as 97 years) and in providing value to Spanish speakers and people who are negatively impacted by social determinants of health (who may also be encountering health disparities in outcomes and health access issues relating to COVID-19). We are unable to present detailed results due to client confidentiality restrictions. Broadly, we measured the number of views of the content, the satisfaction survey responses, and the opt-out rates. These metrics suggest robust engagement with this material across audience groups.

Beyond COVID-19, our next step is to explore the ongoing use of these types of visual stories to build health literacy and awareness in other contexts. For example, is it necessary to get preventive screenings such as mammograms when you feel healthy and nobody in your family has a history of breast cancer? Similarly, how do you decide whether to use a nurse line, urgent care, or the emergency room when you feel sick after hours? We also want to consider tailoring the fotonovelas based on member demographics (age, gender, language, social determinants of health, health literacy) and psychographics (self-efficacy, health beliefs, stage of change) to build variations within story scenes based on these attributes, beliefs, and preferences. We expect that this type of data-driven and artificial intelligence–enabled segmentation will increase the relevance of the fotonovelas and further deepen engagement. Finally, we expect to build our dataset of member responses so that we can rely more heavily on machine learning–based natural language processing to improve recognition accuracy and response handling. 

We were able to quickly develop and deploy a text messaging and fotonovela outreach in English and Spanish to address concerns and influence behavior relating to the COVID-19 crisis. This program is a cost-effective and convenient solution for building health literacy and engaging with underserved, under-resourced and hard-to-reach populations. Member responses and engagement insights can be used to improve the design of future text and mobile friendly visual story-based solutions.

## Abbreviations

CDC: Centers for Disease Control and Prevention
COVID-19: coronavirus disease
HBM: health belief model
NLU: natural language understanding
